# Supplementary material for: Characterisation of Mid-Gestation Amniotic Fluid Cytokine and Bacterial DNA Profiles in Relation to Pregnancy Outcome in a Small Australian Cohort
Source: Microorganisms. 2023 Jun 29;11(7):1698. doi: 10.3390/microorganisms11071698 (PMC10384451; doi:10.3390/microorganisms11071698)
Supplement: Supplementary file 1 [file microorganisms-11-01698-s001.zip › microorganisms-2419061-supplementary.pdf]

Table S1: Unadjusted mean cytokine levels and their 95% confidence intervals, by presence of each genus

		IL-10		IL-1β		IL-6		MCP-1		TNF-α	
		**Marginal geometric mean**	**p**	**Back-transformed marginal mean**	**p**	**Marginal geometric mean**	**p**	**Marginal geometric mean**	**p**	**Marginal geometric mean**	**p**
Novosphingobium* sp.	**Low**	6.01 (3.73, 9.67)	0.375	0.85 (0.49, 1.30)	0.688	139.20 (60.84, 318.48)	0.211	953.27 (739.17, 1229.38)	0.494	5.13 (4.04, 6.51)	0.230
	**High**	4.29 (2.35, 7.83)		0.73 (0.37, 1.21)		72.27 (37.94, 137.68)		861.17 (734.33, 1009.92)		3.91 (2.66, 5.75)	
**Sphingomonadaceae_ Unclassified**	**Absent**	6.35 (4.03, 10.02)	0.217	0.63 (0.37, 0.97)	0.201	124.53 (62.42, 248.47)	0.413	814.37 (673.17, 985.19)	0.145	4.74 (3.83, 5.87)	0.591
	**Present**	3.95 (2.12, 7.35)		1.01 (0.54, 1.63)		80.79 (35.86, 181.98)		1008.05 (808.69, 1256.56)		4.18 (2.72, 6.41)	
***Ralstonia* sp.**	**Absent**	4.66 (2.58, 8.40)	0.597	0.68 (0.35, 1.12)	0.375	100.29 (47.90, 209.97)	1.000	877.06 (711.03, 1081.84)	0.612	4.29 (3.01, 6.11)	0.669
	**Present**	5.65 (3.62, 8.79)		0.94 (0.55, 1.43)		100.32 (45.74, 220.04)		945.42 (763.65, 1170.46)		4.71 (3.59, 6.18)	
***Sphingomonas* sp.**	**Absent**	4.34 (2.78, 6.78)	0.099	0.60 (0.38, 0.86)	**0.017**	93.47 (49.81, 175.41)	0.608	871.62 (756.30, 1004.53)	0.464	3.94 (3.09, 5.01)	**0.050**
	**Present**	8.48 (4.35, 16.52)		1.56 (0.80, 2.56)		126.46 (45.94, 348.08)		1029.04 (665.63, 1590.86)		6.84 (4.16, 11.26)	
***Pelomonas* sp.**	**Absent**	4.55 (3.01, 6.87)	0.103	0.84 (0.53, 1.20)	0.304	80.48 (46.44, 139.47)	**0.005**	895.61 (753.25, 1064.88)	0.409	4.50 (3.44, 5.90)	0.622
	**Present**	10.22 (4.18, 24.98)		0.54 (0.18, 1.08)		419.75 (158.64, 1110.65)		976.96 (863.86, 1104.86)		4.18 (3.62, 4.82)	

*For *Novosphingobium* sp., values lower than the median (median=0.943) were classified as low relative abundance, and values equal to or greater than the median were classified as high relative abundance.
